# Research of Multicopper Oxidase and Its Degradation of Histamine in *Lactiplantibacillus plantarum* LPZN19

**DOI:** 10.3390/microorganisms11112724

**Published:** 2023-11-08

**Authors:** Huijie Pei, Yilun Wang, Wei He, Lin Deng, Qinjie Lan, Yue Zhang, Lamei Yang, Kaidi Hu, Jianlong Li, Aiping Liu, Xiaolin Ao, Hui Teng, Shuliang Liu, Likou Zou, Ran Li, Yong Yang

**Affiliations:** 1 College of Food Science, Sichuan Agricultural University, Ya’an 625014, China; phj8798@163.com (H.P.); wangyilun7841@163.com (Y.W.); 18227587976@163.com (W.H.); 15539571357@163.com (L.D.); 18402879976@163.com (Q.L.); 18215525624@163.com (Y.Z.); 2021318041@stu.sick.edu.cn (L.Y.); kaidi.hu@sicau.edu.cn (K.H.); jlli999@sicau.edu.cn (J.L.); huavslin@163.com (X.A.); 14864@sicau.edu.cn (H.T.); lsliang999@163.com (S.L.); liran@sicau.edu.cn (R.L.); 2 College of Resource, Sichuan Agricultural University, Chengdu 611130, China; zoulikou@sicau.edu.cn

**Keywords:** histamine, multicopper oxidase, prokaryotic expression, purification, spatial structure, degradation products

## Abstract

In order to explore the structural changes and products of histamine degradation by multicopper oxidase (MCO) in *Lactiplantibacillus plantarum* LPZN19, a 1500 bp *MCO* gene in *L. plantarum* LPZN19 was cloned, and the recombinant MCO was expressed in *E. coli* BL21 (DE3). After purification by Ni^2+^-NTA affinity chromatography, the obtained MCO has a molecular weight of 58 kDa, and it also has the highest enzyme activity at 50 °C and pH 3.5, with a relative enzyme activity of 100%, and it maintains 57.71% of the relative enzyme activity at 5% salt concentration. The secondary structure of MCO was determined by circular dichroism, in which the proportions of the α-helix, β-sheet, β-turn and random coil were 2.9%, 39.7%, 21.2% and 36.1%, respectively. The 6xj0.1.A with a credibility of 68.21% was selected as the template to predict the tertiary structure of MCO in *L. plantarum* LPZN19, and the results indicated that the main components of the tertiary structure of MCO were formed by the further coiling and folding of a random coil and β-sheet. Histamine could change the spatial structure of MCO by increasing the content of the α-helix and β-sheet. Finally, the LC-MS/MS identification results suggest that the histamine was degraded into imidazole acetaldehyde, hydrogen peroxide and ammonia.

## 1. Introduction

Biogenic amines are a general class of bioactive, amino-containing, low molecular mass organic compounds. An appropriate amount of biogenic amines can regulate some physiological functions of the human body; they have been shown to be precursors for the synthesis of hormones, alkaloids, nucleotides, proteins, and aromatic compounds [1,2]. But excessive biogenic amines intake can cause food allergies and food poisoning, including symptoms such as nausea, respiratory distress, hot flashes, sweating, palpitations, headache, rashes, stomatitis, hypertension, and other diseases [3]. Histamine is the most toxic biogenic amine and exists in various kinds of fermented foods, such as soy products, cheese and sausages [4].

At present, there are several methods to reduce the content of histamine by inhibiting histamine formation, which specifically include controlling the content of free histidine [5], inhibiting the reproduction of microorganisms with histidine decarboxylase [6], affecting the activity of histidine decarboxylase [7] and adding histamine-degrading enzymes [8]. However, the most direct way to reduce histamine in food is to add histamine-degrading enzymes to degrade histamine in food [9].

Based on published studies, the enzymes with amine degradation activity mainly include three categories: amine oxidases [10], amine dehydrogenase [11] and multicopper oxidase (MCO) [12]. The degradation of histamine is a multi-step reaction: histamine produces imidazole acetaldehyde, ammonia and water under the action of amine oxidase or amine dehydrogenase. Then, imidazole acetaldehyde was catalyzed by aldehyde dehydrogenase to produce imidazole acetic acid. Imidazole acetaldehyde was further oxidized to carbon dioxide and water [8,13]. However, few studies have reported on the products formed by the degradation of histamine by MCO.

We previously screened a strain of *Lactiplantibacillus plantarum* LPZN19 (GeneBank accession number: MW282954) with a strong histamine degradation ability in fermented sausages [4]. Although our laboratory previously identified the presence of the *MCO* gene in LPZN19, revealing its ability to degrade histamine in sausages, the MCO protein was not studied. Consequently, the enzymatic properties of MCO, the mechanism of degrading histamine, and its products remain uncertain. It is essential to investigate the enzymatic properties of MCO to better comprehend its degradation mechanism and potential application in the food industry.

Therefore, in this study, the MCO was heterologously expressed and purified by the prokaryotic expression technique, and its enzymological properties were investigated. Additionally, to clarify the mechanism by which MCO catalyzes the degradation of histamine, the composite reaction system of the purified MCO and histamine was used as the research object, and the inductive effect of histamine on the MCO spatial structure and degradation products of histamine were examined through spectroscopy and mass spectrometry techniques. This study may provide a theoretical basis for the efficient application of multicopper oxidase in the fermented foods processing industry.

## 2. Materials and Methods

### 2.1. Primers, Plasmid and Strains 

The whole-genome sequence of *L. plantarum* LPZN19 was obtained by whole genome sequencing; the two primers used to amplify the *MCO* gene were sense (5′-tgggtcgcggatccgaattcATGGCAAAAAAAGTCTATACTGATT-3′) and antisense (5′-cgagtgcggccgcaagcttTTACATACCGGGCATCCAA-3′). The pET-28a vector (Novagen, Madison, WI, USA) was used for expression of the *MCO* gene. Genetic tools, such as the start codon, 6 × His-tag and terminator were provided in the pET-28a vector. *E. coli* DH5α and *E. coli* BL21 (DE3) were grown in LB medium at 37 °C, and the medium was supplemented with kanamycin (0.03 mg/mL) when screening the transformants.

### 2.2. Homologous Recombination of the MCO/pET-28a

The cloning strategy was used to amplify the *MCO* gene fragment from *L. plantarum* LPZN19, and then, homologous recombination would be performed. The total DNA was first isolated from *L. plantarum* LPZN19, and then using *MCO* F/R primers with the EcoRI/HindIII site, PCR was conducted following this thermal cycling profile: initial denaturation at 98 °C for 2 min, 30 cycles of 98 °C for 30 s, 55 °C for 30 s, and 72 °C for 30 s, and a final extension step at 72 °C for 1 min. After PCR, the fragment size was initially visualized in 1% agarose gel. Before ligation, the PCR product was purified using a DNA Purification Kit (Tiangen Biotechnology, Beijing, China). To assemble the *MCO*/pET-28a expression plasmid, the recombinant *MCO* and linearized pET-28a plasmid was digested with EcoRI/HindIII. The ligation mixture was transferred into *E.coli* DH5α, and recombinants were screened with 30 μg/mL kanamycin in the LB media. Positive transformants were sent to Sangon Biotech (Sangon Biotech Co., Ltd., Shanghai, China) for gene sequencing.

### 2.3. Heterologous Expression and Purification of Recombinant MCO

The purified ligation product was transformed into *E. coli* BL21 (DE3) and screened with 30 μg/mL kanamycin in LB media. Transformants containing the correct plasmid were validated by plasmid isolation and EcoRI/HindIII double-enzyme digestion.

The expression of MCO was initiated by two steps. Firstly, the *E. coli* BL21 (DE3) host was cultured in LB medium supplemented with 30 μg/mL kanamycin at 37 °C for 6 h. Then, the host induced was performed with 1 mmol/L IPTG and 0.5 mmol/L CuCl_2_ at 25 °C for 14 h. Cells were centrifugally collected and then lysed by ultrasound on ice to fully lyse host cells. MCO was finally purified by Ni^2+^-NTA agarose affinity chromatography (1.6 × 5 cm, Qiagen, Hilden, Germany), and eluted samples were concentrated by polyethylene glycol 20,000. The SDS-PAGE was used to analyze the expression and purification processes [14]. Then, the specific activity and fold purification were calculated by enzyme activity.

### 2.4. Determination of the Enzymatic Properties of Recombinant MCO

#### 2.4.1. Measurement of Enzyme Activity 

The activity of MCO was determined by the visible light absorption method [15]. Different buffers were prepared: 50 mmol/L MES buffer (pH 2.5, 3.0, 3.5, 4.0, 4.5, 5.0). In the mixed reaction system of 0.1 mL 0.49 mg/mL MCO and 1.9 mL 50 mmol/L of the above buffer containing 1.0 mmol/L 2, 2′-azino-bis (3-ethylbenzothiazoline-6-sulfonic acid) (ABTS), the activity of MCO was expressed by detecting the oxidation amount of ABTS (reaction time 2 h). Enzyme activity was defined as the amount of enzyme required to oxidize 1 µmoL ABTS per minute was defined as an enzyme activity unit (U).

#### 2.4.2. The Effect of pH on the Activity and Stability of Recombinant MCO 

The pH of the reaction system was adjusted to 2.5, 3.0, 3.5, 4.0, 4.5, and 5.0. Then, the enzyme activity was determined at the optimum pH after 2 h, and the pH with the highest relative enzyme activity was considered the optimum condition. The purified recombinant MCO was incubated in MES buffer (pH 2.5–5.0) at 4 °C for 60 min, and the remaining enzyme activity was determined and relative enzyme activity was calculated by setting the conditions for the highest enzyme activity, which was defined as 100%.

#### 2.4.3. The Effect of Temperature on the Activity and Stability of Recombinant MCO 

The activity of MCO was investigated at different temperatures. The reaction system was incubated at 40 °C, 45 °C, 50 °C, 55 °C, 60 °C, 65 °C, and 70 °C for 2 h to determine the enzyme activity, and the temperature with the highest relative enzyme activity was considered the optimum condition. The purified recombinant MCO was incubated in MES buffer (optimum pH) at 40–65 °C for 60 min, and the remaining enzyme activity was determined and relative enzyme activity was calculated by setting the conditions for the highest enzyme activity, which was defined as 100%.

#### 2.4.4. Determination of Kinetic Parameters of Recombinant MCO 

Different concentrations of substrate (0.1–1 mmol/L ABTS) solutions were mixed with purified MCO, respectively. Then, the reaction was carried out at the optimum reaction pH and temperature for 2 h, and the enzymatic activity of recombinant MCO was determined. The Michaelis constant (*K*m) and maximal activities (*V*max) value of recombinant MCO were calculated according to the Lineweaver–Burk’s double reciprocal plotting method.

#### 2.4.5. Effect of Metal Ions on Recombinant MCO Activity

The enzyme solution was mixed with MES buffer containing 1 mmol/L metal ions (Ca^2+^, Fe^2+^, Cu^2+^, Zn^2+^, Mg^2+^, Mn^2+^, Ni^2+^), and the enzyme activity was determined after 2 h under optimum conditions.

#### 2.4.6. Effect of Sodium Chloride on Recombinant MCO Activity

NaCl was added to the purified enzyme solution at final concentrations of 0%, 10%, 15%, 18% and 20% (*w*/*v*), respectively. And the enzyme activity was measured after reacting for 2 h at optimum temperature and optimum pH.

#### 2.4.7. Investigation of the Substrate Specificity and Optimal Substrate of Recombinant MCO

Mixed enzyme solutions (100 mmol/L) containing cadaverine, putrescine, tyramine, histamine, phenylethylamine, tryptophan and spermidine were prepared, respectively. The reaction system was reacted for 12 h at the optimum temperature and pH; then, a boiling water bath was used to terminate the reaction, and the enzyme activity was measured.

### 2.5. Identification and Prediction of Recombinant MCO Structure

#### 2.5.1. Identification of the Secondary Structure 

The identification method of the protein secondary structure was as follows [16]: the test solution was placed in a 1 mm quartz absorption cell with a scanning speed of 60 nm/min, and the test band was 190–260 nm. With 10 mmol/L buffer as the solvent background, the sample and background were injected into a colorimetric dish for testing and scanning, respectively.

#### 2.5.2. Prediction of the Tertiary Structure 

The SWISS-MODEL (https://swissmodel.expasy.org/interactive, accessed on 15 March 2021) network software was used for homology modeling and to predict the three-dimensional structure model [17].

### 2.6. The Change in Spatial Structure after MCO Acting on Histamine

#### 2.6.1. Preparation of the Histamine–MCO Reaction System 

Histamine–MCO mixed reaction systems were configured with histamine concentrations of 0, 50, 100, 200, 400, and 800 mg/L, respectively. Then, the reaction system was placed in an incubator at 50 °C for 24 h and used for subsequent experiments.

#### 2.6.2. Determination of Ultraviolet Spectrum and Endogenous Fluorescence Spectra

The changes of secondary structure of histamine treated by MCO were characterized by UV absorption spectra and endogenous fluorescence spectra. A UV-visible spectrophotometer (Multiskan SkyHigh, Thermo Fisher Scientific Inc., Waltham, MA, USA) was used to scan the composite system prepared, and the scanning range was 200–400 nm [18]. The determination of endogenous fluorescence spectra was made according to the method of Dankowska and Kowalewski [19]: the emission wavelength was 300–500 nm, the excitation wavelength was 290 nm, the width of the emission and excitation slit was 10.0 nm, and the voltage was 550 mV.

#### 2.6.3. Determination of Secondary Structure Change in MCO

The circular dichroism of the composite system was determined according to the method of Wu et al. [16]. The spectral scanning range was 200–250 nm, the scanning rate was 100 nm/min, and the determination was carried out at room temperature. The changes of α-helix, β-sheet, β-turn and random coil content in each secondary structure were calculated by the CONTIN/LL method.

#### 2.6.4. Characterization of the Tertiary Structure Changes of MCO

The surface hydrophobicity was represented by the fluorescence intensity of 1-anilinonaphthalene-8-sulfonic acid (ANS) to characterize the change in MCO tertiary structure. The excitation wavelength was 390 nm, and the excitation and emission slits were 5.0 nm and 2.5 nm, respectively. Then, 20 µL of ANS (90 mmol/L) was added to 4 mL of the complex system and mixed well, which was followed by the determination of surface hydrophobicity [20,21].

### 2.7. Monitoring and Identification of Histamine Degradation Products 

The histamine–MCO reaction system was configured according to the configuration group in Section 2.6.1. To avoid false positive results, the enzyme concentration was doubled during product identification to reduce the substrate consumption time and prevent prolonged sample detection. The final concentrations of histamine and MCO in the system were 500 mg/L and 1 mg/mL, respectively. The temperature of the reaction system was 50 °C, and the samples were analyzed at different times. The degradation process of histamine was detected by a Liquid Chromatograph Mass Spectrometer (Liquid Chromatography: Waters I-CLASS (Milford, MA, USA), Mass spectrometer: Waters XEVO-TQS micro, Chromatographic column: ACQUITY UPLCr BEH C18 1.7 µm, Column temperature: 25 °C) [4]. The amount of hydrogen peroxide and ammonia in the system was determined using a Hydrogen Peroxide Assay Kit (Abcam Trading Co., Ltd., Shanghai, China) and Ammonia Assay Kit (Abcam Trading Co., Ltd., Shanghai, China).

### 2.8. Statistical Analysis 

The experimental data were analyzed using IBM SPSS Statistics 25.0 (IBM, Armonk, NY, USA) in triplicate. One-way analysis of variance (ANOVA) was applied to analyze the significances between groups, and significant difference was considered when *p* < 0.05.

## 3. Results 

### 3.1. Sequence Analysis of the MCO

**Figure 1:**
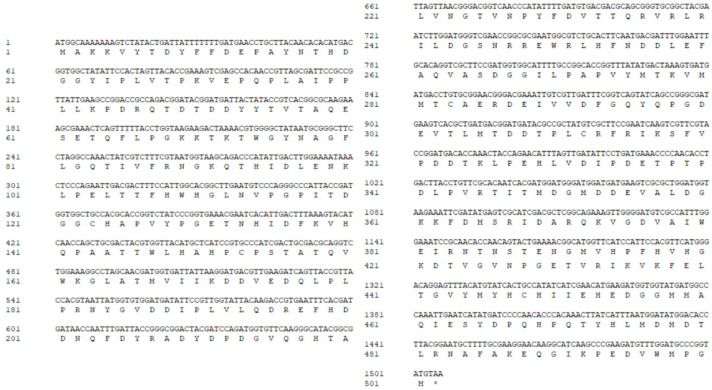
DNA sequence and amino acid sequence of MCO. The termination codon (TAA) was marked with an asterisk (*).

The *MCO* gene in *L. plantarum* was amplified by PCR and cloned into the pET-28a vector of *E. coli* DH5α. The *MCO* gene sequence and its encoded amino acid sequence are shown in Figure 1. MCO contains 501 amino acid residues, there was no signal peptide and transmembrane structure, and it contains two domains (57–175 amino acids and 336–468 amino acids), one of which is a type III (coupled dinuclear) MCO domain (57–175 amino acids). The predicted molecular weight and theoretical isoelectric point (PI) of MCO were 56.8 kDa and 4.93, respectively. Therefore, our cloned *MCO* encodes a mature peptide of MCO.

### 3.2. Heterologous Expression and Purification of Recombinant MCO

**Figure 2:**
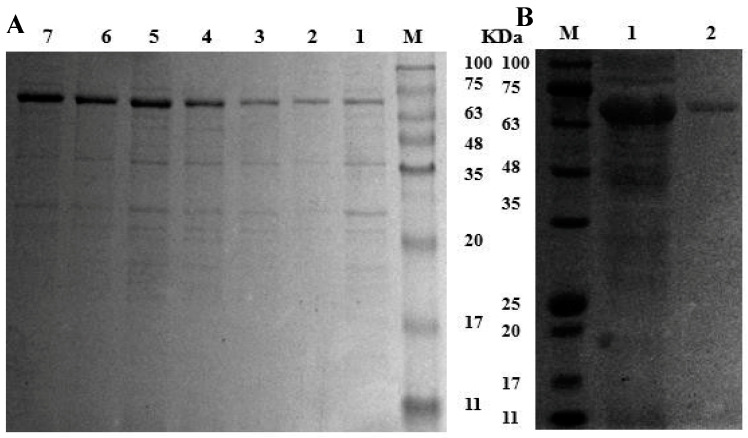
Construction of recombinant plasmid and expression of MCO. (**A**) Expression of MCO in *E. coli* BL21 (DE3). M: Protein Marker; 1–7: The bacterial proteins of recombinant bacteria that were induced for 2, 4, 6, 8, 10, 12, and 14 h, respectively. (**B**) Purification of recombinant proteins. M: Protein Marker; 1: Total proteins; 2: The purified MCO.

The genomic DNA of *L. plantarum* LPZN19 was used as a template to amplify MCO. The recovered MCO and the linear pET-28a, after double enzyme digestion and recovery, were ligated and transformed into the clone host *E. coli* DH5α. The plasmid extracted from the positive transformants (verified by colony PCR) was sent to Sangon Biotech for sequencing, where the successful construction of the recombinant plasmid *MCO*/pET-28a was verified. The recombinant plasmid *MCO*/pET-28a was transformed in the expression host *E. coli* BL21 (DE3). The constructed recombinant strains were induced to express for 2, 4, 6, 8, 10, 12, and 14 h, respectively. Then, cells were collected and disrupted. Samples collected during the processes of expression and purification were analyzed by SDS-PAGE (Figure 2). It was observed that the protein band intensity increased with the extension of expression time at about 67 kDa (Figure 2A). After detection by mass spectrometry, the molecular weight of the recombinant MCO was 57.10 kDa (Table 1), which was consistent with the theoretical prediction. The observed difference in molecular weight on SDS-PAGE may be attributed to the presence of approximately 7.0 Kda promoter protein and histidine-labeled protein. Compared to the crude enzyme solution, the purified MCO by the Ni^2+^-NTA column had a single band (Figure 2B). The recovery of purified recombinant MCO was about 31.89%, the fold purification was 6.91, and the enzyme activity was 60.75 U/L (Table 2). 

### 3.3. Effects of Physicochemical Factors on Recombinant MCO Activity

**Figure 3:**
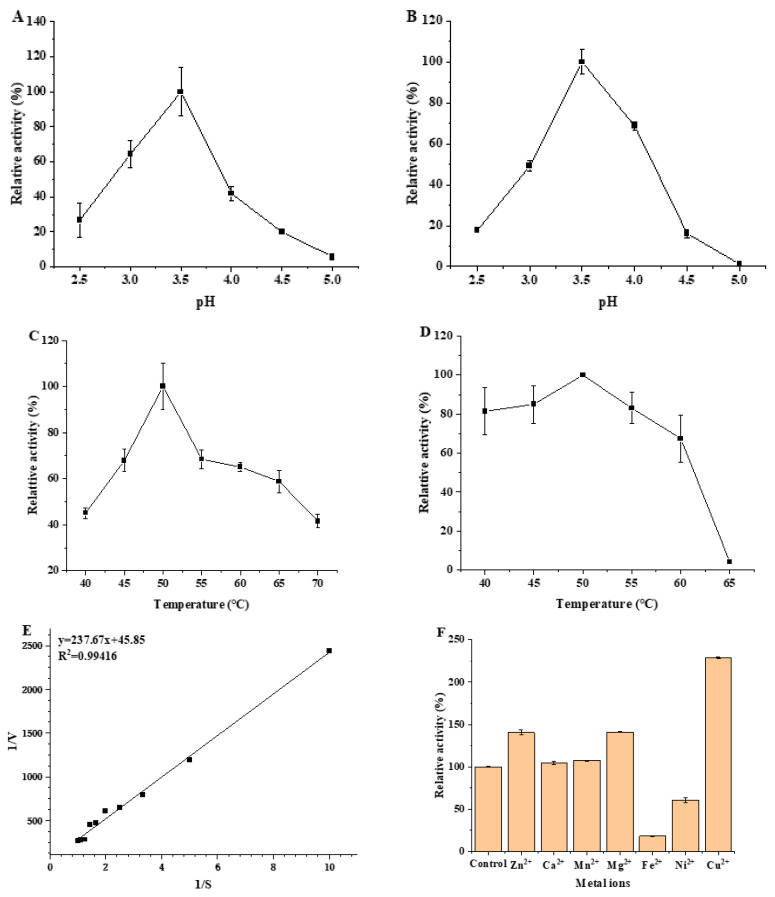

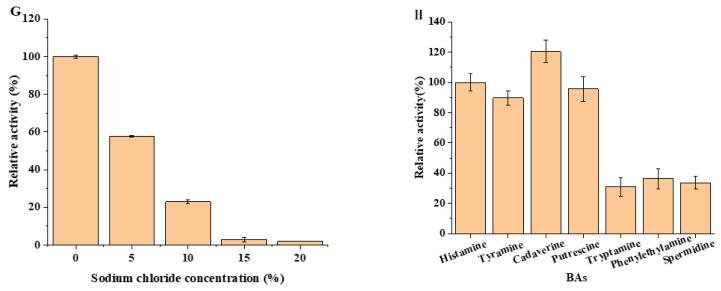
Determination of recombinant MCO enzymatic properties. (**A**) Optimum reaction pH value of recombinant MCO. (**B**) The acid–base stability of recombinant MCO. (**C**) Optimum reaction temperature of recombinant MCO. (**D**) The thermal stability of recombinant MCO. (**E**) Kinetic parameters for recombinant MCO. (**F**) Effect of metal ions on the activity of recombinant MCO. (**G**) Effect of sodium chloride on the activity of recombinant MCO. (**H**) The optimal substrate of recombinant MCO.

The main factors affecting MCO activity include pH, temperature, salinity, metal ions, etc. Exploring the specific effects of these factors on enzyme activity could provide insight into the enzymatic properties of MCO, which is one of the important means to eliminate biogenic amines in meat products by using MCO. Therefore, the enzymatic properties of MCO were explored using ABTS as the reaction substrate, and the results are presented in Figure 3.

#### 3.3.1. Optimum pH and pH Stability of MCO

The effects of pH and acid–base stability on MCO enzyme activity were investigated according to the change in pH during sausage fermentation (pH 4.5–5.5), and the results are shown in Figure 3A,B. The enzyme activity of MCO showed a strong dependence on pH; the effect of pH on the activity ranged pH from 2.5 to 5.0 (Figure 3A). Maximum activity of the MCO was obtained at pH 3.5, and the maximum activity was considered 100%. Specifically, in the pH range of 2.5–3.5, the enzymatic activity was proportional to the pH. When the pH > 3.5, the enzyme activity became relatively low, and there was an inactivation of enzyme activity at pH 5.0. The results of the study have shown that the optimal pH value for MCO enzyme activity was 3.5. The results of acid–base stability on MCO enzyme activity showed a similar trend to the results with pH (Figure 3B). The enzyme activity was relatively stable in the pH range of 3.0–4.0, and the remaining relative enzyme activities were all above 45%. The optimum reaction pH of MCO and the results of the acid–base stability study demonstrated that MCO is an acidic enzyme with great potential for application in fermented foods.

#### 3.3.2. Optimum Temperature and Thermal Stability of MCO

In terms of application, temperature is one of the key environmental parameters for enzyme activity. The effects of optimal temperature and thermal stability on MCO enzyme activity were investigated, and the results are shown in Figure 3C,D. The activity of MCO showed an initial increase that was followed by a decrease with the temperature change, and the activity reached a maximum at 50 °C, indicating that the optimal temperature for the MCO reaction was 50 °C (Figure 3C). To be specific, the enzyme activity increased rapidly with the increase in temperature in the range of 40–50 °C, and the activity first decreased rapidly (50–55 °C) and then decreased gradually (55–70 °C). Furthermore, MCO still retained more than 40% of the enzyme activity at the temperature of 40–70 °C, suggesting that MCO has excellent temperature resistance in a range from 40 to 70 °C. In terms of thermal stability, MCO is less affected by temperature changes compared to acid–base stability (Figure 3D). The activity of MCO was relatively stable in the temperature range of 40–60 °C, and the observed residual enzyme activity was maintained above 60%, indicating that MCO has excellent thermal stability. 

#### 3.3.3. Determination of Kinetic Parameters of MCO 

The dependence of the reaction rate on the concentration of ABTS in the system showed a typical Michaelis–Menten kinetics (Figure 3E). The *K*m, *K*cat, *K*cat/*K*m and *V*max values of MCO were found to be 2.18 ± 0.13 mmol/L, 3.77 ± 0.09 s^−1^, 1.73 ± 0.16 mmol/L·s, and 9.96 × 10^−2^ ± 0.15 mmol/(L·min), respectively(Table 3). The *K*m value of MCO obtained from *L. plantarum* LPZN19 was lower than that of *Klebsiella* sp. (5.63 mmol/(L·min)) [22], while it was higher than that of *Ochrobactrum* sp. (0.072 mmol/(L·min)) [23]. Consequently, there is variation in the binding affinity of MCOs obtained from different strain sources for the substrate.

#### 3.3.4. Effect of Metal Ions on Recombinant MCO Activity 

The results showed that Zn^2+^, Mg^2+^, and Cu^2+^ could increase the activity of recombinant MCO, and the enzyme activity was increased by 40.60%, 40.91%, and 128.48%, respectively (Figure 3F). Therefore, Cu^2+^ had the most significant activating effect on recombinant MCO. The addition of Cu^2+^ (1 mmol/L) to the blank group increased the enzyme activity of MCO by 2.3 times, which may be related to the four Cu^2+^ binding sites in MCO [22]. Additionally, Ca^2+^ and Mn^2+^ did not affect the MCO enzyme activity, while Fe^2+^ and Ni^2+^ inhibited the enzyme activity.

#### 3.3.5. Effect of Sodium Chloride on Recombinant MCO Activity

Since a certain concentration of sodium chloride was added in fermented sausage, it was necessary to investigate the effect of sodium chloride on MCO enzyme activity. The activity revealed low enzymatic activity of MCO deceasing with the increase in concentration of NaCl in the mixed reaction system (Figure 3G). Concretely speaking, after the addition of 5, 10%, and 15% NaCl, the observed residual activities were 57.71%, 22.93% and 2.81%, respectively. The activity was almost completely lost under the condition of 15% NaCl, indicating that the enzyme can tolerate up to this salt concentration. Importantly, when the NaCl concentration was 5%, the enzyme activity remains larger than 50%, while the salt addition in sausages is generally 3%, suggesting that the enzyme plays an active role in the high salt environment during the maturation, drying, and storage of fermented sausages.

#### 3.3.6. Degradation of Biogenic Amine by MCO

In order to explore the substrate specificity and the optimal substrate of MCO, the degradation of seven biogenic amines in food by MCO was tested under the optimal conditions. The MCO enzyme activity for histamine degradation was defined as 100%, and the relative enzyme activity of MCO for degrading other biogenic amines was calculated. The results are presented in Figure 3H. Among the seven biogenic amines tested, MCO exhibited excellent degradative capacity with relative enzyme activities of 120.44%, 100%, 95.61% and 89.69%, respectively. This indicated that MCO has no substrate specificity and can effectively degrade the four major biogenic amines in fermented sausage.

### 3.4. Identification of the Structure of Recombinant MCO 

#### 3.4.1. Identification of the Secondary Structure 

**Figure 4:**
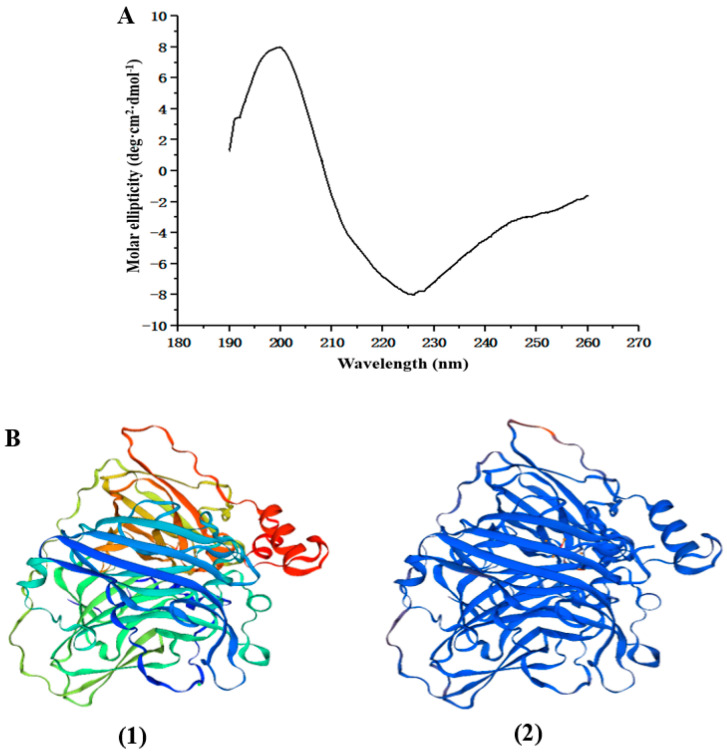
Measurement and prediction of recombinant MCO spatial structure. (**A**) Determination of secondary structure of MCO. (**B**) Prediction of tertiary structure of MCO; (**1**) the tertiary structure of template protein; (**2**) the tertiary structure of MCO.

The secondary structure of MCO was analyzed by circular dichroism. The circular dichroic spectrum of MCO in the far ultraviolet region (190–260 nm) is shown in Figure 4A. A strong positive peak appears at 198 nm, which was the characteristic peak of β-folding. The data were imported into CD pro software (https://www.colostate.edu, accessed on 17 March 2021) to predict the proportion of the secondary structure. The distribution of the four secondary structures in MCO was as follows: 2.9% α-helix, 39.7% β-sheet, 21.2% β-turn, and 36.1% random coil.

#### 3.4.2. Prediction of the Tertiary Structure 

The tertiary structure of MCO was simulated by SWISS-MODEL online software. The MCO sequence of *Pediococcus pentosaceus* (Code 6xj0.1.A in the Protein Data Bank) has a similarity of 68.21% with the MCO of *L. plantarum*, indicating that the MCO produced by the two strains had homology [14]. Therefore, the MCO of *P. pentosaceus* was used as a template protein to predict the MCO of *L. plantarum* in this study (Figure 4B). The tertiary structure of MCO was mainly composed of a random coil and β-sheet further curling and folding through the interaction of side-chain groups. This finding was consistent with the results obtained after the secondary structure was determined.

### 3.5. Analysis on the Change in Recombinant MCO Spatial Structure

Although most MCOs have similar catalytic center structures, their biological functions and catalytic performance vary widely [24]. Therefore, exploring the enzymatic properties of the MCO is the basis for studying its structure and mechanism of action, which is of great significance to its biological field.

#### 3.5.1. Determination of Structure Induction of MCO by Histamine 

**Figure 5:**
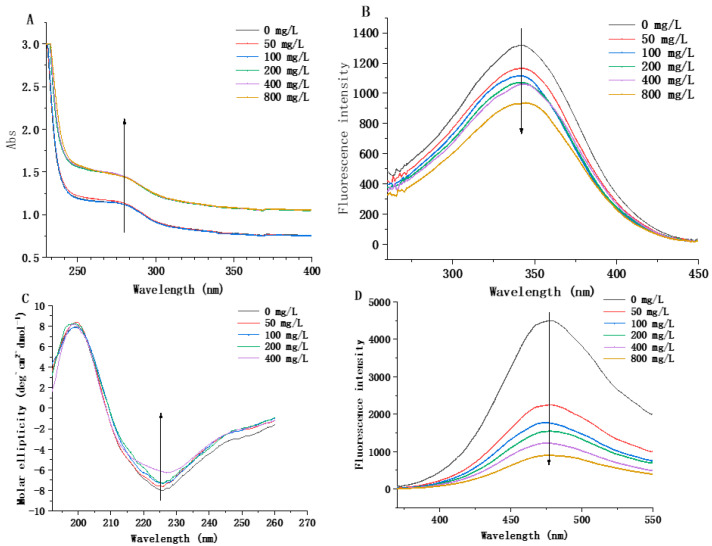
Changes in spatial structure of recombinant MCO. (**A**) Ultraviolet spectrum of the different concentrations of histamine and MCO reaction system. (**B**) Endogenous fluorescence spectra of the different concentrations of histamine and MCO reaction system. (**C**) Circular dichroism spectra of the different concentrations of histamine and MCO reaction system. (**D**) ANS fluorescence spectra of the different concentrations of histamine and MCO reaction system.

UV absorption spectroscopy can effectively detect structural changes and intermolecular interactions [25]. Generally, in UV absorption spectra, the peak values of proteins at 250 nm and 280 nm occur mainly due to the presence of phenylalanine amino acid residues and two aromatic amino acids (tyrosine and tryptophan) [26]. After the addition of histamine, the absorption peak of the wavelength of the composite system increased significantly from 1.12 to 1.44 as the concentration of histamine increased, and the absorbance peak shifted from 280 to 277 nm with a slight blue shift (Figure 5A). The addition of histamine might cause the extension of the peptide chain, resulting in an increase in the chromophoric groups in the molecule and an increase in the UV absorption intensity. The above results indicated that the spatial structure of MCO changed. The conformational changes of the proteins were then further investigated by fluorescence spectroscopy (Figure 5B). With the increase in histamine concentration, the fluorescence peak intensity gradually decreased (at 340 nm), indicating that the histamine had a quenching effect on the endogenous fluorescence of MCO, thus changing the spatial conformation of MCO. This result was consistent with the result of UV scanning.

#### 3.5.2. Changes in Secondary Structure of Recombinant MCO 

The circular dichroism of the composite system was measured, and the results are shown in Figure 5C. In the range of histamine concentration of 0–200 mg/L, the amplitude of the composite system’s peak at 225 nm exhibited a slight reduction from 7.96 to 7.20. Furthermore, the amplitude of the composite system’s peak at 225 nm was decreased significantly from 7.20 to 6.11 with the increase in histamine concentration. This indicated that the stability of the secondary structure of MCO was weakened after the binding of histamine to MCO, which was consistent with the UV scanning results. Furthermore, with the increase in histamine concentration in the composite system (0–400 mg/L), the α-helix content in MCO increased by 0.3%, the β-sheet content increased by 2.1%, and the random coil decreased by 2.5% (Table 4).

#### 3.5.3. Changes in Tertiary Structure of Recombinant MCO

The hydrophobic interaction between the nonpolar side chains of amino acid residues in proteins is the most important force in maintaining the tertiary structure of proteins, which can be indirectly revealed by measuring the amount of hydrophobic interaction between ANS (a nonpolar substance) and proteins [27]. Fluorescence spectroscopy with ANS as the fluorescent probe was used to determine the change in the hydrophobicity on the MCO surface due to the action of histamine at different concentrations. The results are shown in Figure 5D, where the fluorescence intensity of the composite system decreased with an increase in the histamine concentration. Additionally, the maximum fluorescence absorption peak of the composite system has a slight blue shift (from 479 to 477 nm), indicating that the tertiary structure of MCO changed. This may be because ANS can bind to the hydrophobic region of MCO through non-covalent interactions, and histamine binds to the hydrophobic groups on the surface of MCO, reducing the hydrophobic region of the protein and its binding to ANS, resulting in a decrease in the hydrophobicity of the measured surface [28]. 

### 3.6. Monitoring and Identification of Histamine Degradation Products

**Figure 6:**
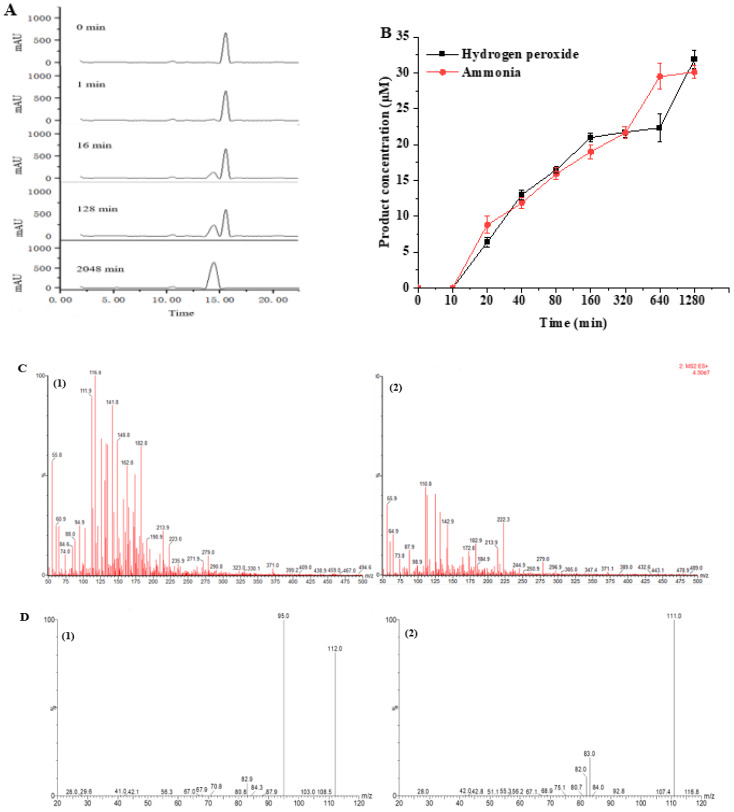
Characterization of histamine degradation products by recombinant MCO. (**A**) Dynamic formation process of histamine degradation products. (**B**) Determination of hydrogen peroxide and ammonia content. (**C**) Primary mass spectra (horizontal axis: *m*/*z*, vertical axis: relative abundance). (**1**) Histamine. (**2**) Imidazole acetaldehyde. (**D**) Secondary mass spectra (horizontal axis: *m*/*z*, vertical axis: relative abundance). (**1**) Histamine. (**2**) Imidazole acetaldehyde.

The dynamic monitoring process of histamine degradation by MCO and the identification of its degradation products are shown in Figure 6. The histamine degradation liquid chromatogram is shown in Figure 6A. When the reaction time was 0 min, only histamine was present, and its peak time was 15.53 min. When the reaction time was reacted for 10 min, a new peak was detected at the peak time of 14.43 min, and the signal of this peak gradually increased with the extension of the reaction time (*K*m = 2.07 ± 0.18 mmol/L), indicating that the substance corresponding to the peak was the degradation product of histamine. Therefore, we also determined the content of hydrogen peroxide and ammonia in the reaction system, which confirmed that the reaction produced those two substances (Figure 6B).

**Figure 7:**
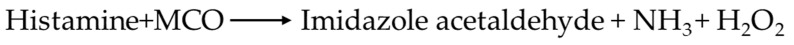
Reaction equation for degradation of histamine by MCO.

The LC/MS data of histamine and its degradation product are presented in Figure 6C. The molecular weights of the two substances were (1) (*m*/*z* 112) and (2) (*m*/*z* 111), respectively. The measured parent nucleus of histamine was consistent with its actual molecular weight of 111 Da, which also confirmed that the molecular weight of the degradation product was 110 Da. To further identify the histamine degradation product, we identified histamine and its degradation products by LC-MS/MS (Figure 6D). The nuclear mass ratio of substance (2) (*m*/*z* 111) was selected as the parent nucleus, and secondary mass spectrometry was performed with the appropriate collision energy. The mass transitions and the corresponding cone voltages and collision energies are presented in Table 5. The mass-to-charge ratios of histamine and imidazole aldehyde product ions were 94.4 and 82.9, which were consistent with the size and type of fracture of the product ions in the identification of histamine and imidazole aldehyde [29]. This confirmed that the degradation product of histamine was imidazole aldehyde, and the catalyzed reaction equation is shown in Figure 7.

## 4. Discussion

In recent years, there have been frequent incidents of histamine poisoning in food, attracting consumers’ attention. Therefore, it is necessary to control the content of histamine in fermented foods. Adding amine-degrading enzymes to food can directly degrade biogenic amines in food without affecting the nutritional composition of food or the flavor of food, which is the most promising method to reduce biogenic amines in food [30]. Multicopper oxidase (MCO) is a class of metal oxidases widely found in plants, animals and microorganisms, including ascorbate oxidase, ceruloplasmin and laccase, and many other types [31]. Compared with other types of amine oxidase and amine dehydrogenase, MCO has no substrate specificity and shows high degradation activity compared to several common biogenic amines in fermented foods [32]. Therefore, it is important to study MCO to reduce the content of histamine and other biogenic amines in food. 

In this study, we described the cloning, expression, and characterization of MCO from the *L. plantarum* LPZN19, whose main feature was the ability to degrade histamine, tyramine, cadaverine, and putrescine. The *L. plantarum* LPZN19 MCO contains two conserved regions with unique copper-binding sites, which are typical structural features of MCOs [33]. Correspondingly, *L. plantarum* LPZN19 MCO was predicted to be 56.85 kDa. Predicted molecular weights of MCO have been reported in *Acinetobacter baumannii* [34] (72 KDa), *Klebsiella pneumoniae* [35] (60 KDa), *Pseudomonas* sp. [15] (52 KDa), *Klebsiella* sp. [22] (58.2 KDa) and *Ochrobactrum* sp. [23] (57.8 KDa). Therefore, the molecular weight of MCO varies among various sources.

The enzymatic properties’ results indicate that MCO has optimal conditions of a pH of 3.0 and temperature of 50 °C, respectively. In addition, enzyme activity can be retained at a level of over 20% when subjected to medium to high temperature conditions (40–60 °C) as well as acidic surroundings (pH 3.0–4.0). These findings reveal that MCO demonstrates remarkable heat resistance, acid resistance, and acid–base stability, which is consistent with the acidic environment and slightly neutral pH that typically favor MCO’s oxidation reactions [22,36]. Furthermore, *L. plantarum* LPZN19 MCO exhibits higher acid–base stability and lower thermal stability than other bacterial MCOs [37]. Metal ions are crucial to the activity of MCO. The enzymatic activity of MCO can be greatly promoted by Cu^2+^, Zn^2+^, and Mg^2+^, and Cu^2+^ is the metal ion-dependent for MCO to maintain enzymatic activity, which is consistent with the majority of the current studies of MCO [24]. Meanwhile, MCO enzymatic activity is inhibited by NaCl, and *L. plantarum* LPZN19 MCO could tolerate the high salt environment of 10% NaCl. This could be because Na^+^ and Cl^−^ impede the binding of protein to substrate [38,39]. In addition, MCO showed the highest degradation activity for cadaverine in fermented foods, which is followed by histamine, putrescine, and tyramine in that order, indicating that MCO is not substrate specific [40]. Therefore, MCO can be applied in different types of food depending on its different enzymatic properties and oxidation capacity, thus offering a wide range of applications.

The function of protein is closely related to its secondary structure, domain, and tertiary structure, and the secondary structure is the basis of the protein tertiary structure [41]. Therefore, it is necessary to elucidate the secondary structure and tertiary structures of proteins to determine their function. Through the determination of circular dichroism and the prediction of the SWISS-MODEL, we found the MCO to be mainly composed of a random coil and the β-sheet. Then, we studied the induction effect of histamine on the MCO structure. Changes in the environment where proteins and other macromolecules are located can lead to changes in their conformational structures, thus leading to changes in the position of chromophoric groups in proteins. This causes changes in the absorption spectra (a decrease or increase in the fluorescence signal strength and a red or blue shift of the maximum emission wavelength) [42,43]. The results of UV spectroscopy, fluorescence spectroscopy, surface hydrophobicity, and circular dichroism showed that the secondary structure and tertiary structure of MCO changed partially with an increase in the histamine concentration, indicating that the addition of histamine can induce the structure of MCO, leading to an interaction between them.

MCO represents a diverse family of enzymes that catalyze the oxidation of a wide range of substrates (such as aromatic polyphenols, non-phenolic substrates and some cyanide complexes of metals), and different MCOs oxidize various substrates and form distinct products [36,44]. Therefore, the products formed by the oxidation of histamine by *L. plantarum* LPZN19 MCO were identified for the first time. The degradation process and degradation products of histamine were monitored and identified by LC-MS/MS. Consistent with biogenic amines oxidase and the biogenic amines dehydrogenation enzyme, the degradation products of histamine by MCO were imidazole acetaldehyde, hydrogen peroxide, and ammonia; this is similar to the results of Sumner and Taylor [45]. Similarly, Sitthisak et al. [46] characterized that the MCO from *S. aureus* has the ability to catalyze substrates, generating H_2_O_2_ in the presence of heavy metals. Some studies showed oxidases or amine dehydrogenase, and the latter is further oxidized to imidazole acetic acid by aldehyde dehydrogenase [8,36]. In this study, histamine was not oxidized to imidazole acetic acid, suggesting that the aldehyde dehydrogenase gene may not be present in *L. plantarum* LPZN19.

Due to the lack of substrate specificity, MCO has the ability to degrade a wide range of biogenic amines that were present in different types of fermented foods [47]; it suggests that MCO has great potential for application in reducing biogenic amines contamination in foods. The research in this paper has a certain reference value for industrial production and application, but there are still some problems and deficiencies, such as the catalytic stability of MCO and the high cost expression level of purification, which restrict its industrial application [36,48]. Therefore, the molecular modification, chemical modification or immobilization of MCO to improve its resistance to extreme conditions and chemical reagents and the realization of MCO recycling have promising prospects for research and development. In addition, the ammonia produced by MCO in degrading histamine should be considered. Therefore, the application of MCO may require a combination of various factors.

## 5. Conclusions

In summary, we obtained MCO by the prokaryotic expression system and purified the recombinant protein by Ni^2+^-NTA affinity chromatography. The MCO is a typical metal oxidase with excellent heat resistance, acid resistance, and strong degradation ability to histamine. In the mixed system of MCO and histamine, the MCO degraded histamine into imidazole acetaldehyde, hydrogen peroxide and ammonia by reducing the stability of the secondary and tertiary structures. This study provides a reference for the mechanism of histamine degradation by adding degradation enzymes in fermented meat products, which can further promote the healthy development of the fermented meat industry.

## Figures and Tables

**Table 1:** Objective protein identification results.

Protein Name	PI	MW (kDa)	Peptide Sequence
MCO	4.93	57.10	EFHDDNQFDYR GLATMVIIKDDVEDOLPLPR EFHDDNQFDYR

Note: MCO: Multicopper oxidase, PI: Isoelectric point, MW: Molecular weight.

**Table 2:** The evaluation of the effect of purification of MCO.

Sample	Volume (mL)	Total Enzyme Activity (U)	Amount of Protein (mg)	Specific Activity (U/mg)	Recovery (%)	Purification Fold
Before purification	85	7.62	362.95	0.02	100	1
After purification	40	2.43	19.6	0.12	31.89	6.91

Note: MCO: Multicopper oxidase.

**Table 3:** Enzyme kinetic parameters of MCO.

Enzyme	pH	*K*m (mmol/L)	*K*cat (s^−1^)	*K*cat/*K*m (mmol/L·s)
MCO	3.5	2.18 ± 0.13	3.77 ± 0.09	1.73 ± 0.16

Note: MCO: multicopper oxidase.

**Table 4:** Changes of secondary structure content of MCO.

	Histamine Concentration (mg/L)				
	0	50	100	200	400
Alpha-helix (%)	2.9 ± 0.02	3.0 ± 0.01	2.9 ± 0.02	3.1 ± 0.03	3.2 ± 0.01
Beta-folding (%)	39.7 ± 0.15	39.9 ± 0.24	40.1 ± 0.17	40.4 ± 0.26	41.8 ± 0.22
Beta-turn (%)	21.2 ± 0.12	21.3 ± 0.08	21.2 ± 0.15	21.2 ± 0.04	21.3 ± 0.09
Random coil (%)	36.1 ± 0.14	35.7 ± 0.31	35.7 ± 0.27	35.2 ± 0.22	33.6 ± 0.19

Note: MCO: multicopper oxidase.

**Table 5:** Mass transitions and corresponding CE and cone voltages.

Compound Name	Molecular Formula	Precursor Ion (*m*/*z*)	Production (*m*/*z*)	Mass Transition	CE (KV)	Cone Voltage (V)
Histamine	C5H9N3	112	94.4	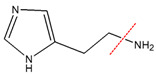	11	30
Imidazole acetaldehyde	C5H6N2O	111	82.9	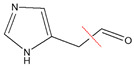	15	30

Note: CE: Collision energies.

## Data Availability

Data available on request due to restrictions privacy: The data presented in this study are available on request from the corresponding author.
